# Metagenomes from untreated wastewater and the soil irrigated with it for 50 years in the Mezquital Valley, Mexico

**DOI:** 10.1128/mra.01299-25

**Published:** 2026-07-23

**Authors:** Eduardo J. Aguilar-Rangel, Kathia Lüneberg, Daniel A. Medina, Christina Siebe, Rocio J. Alcántara-Hernández, Luis E. Servín-Garcidueñas

**Affiliations:** 1 Laboratorio de Microbiómica, Escuela Nacional de Estudios Superiores Unidad Morelia, UNAM 199806, Morelia, Michoacán, México; 2 Instituto de Geología, Ciudad Universitaria, UNAM, Ciudad de México, México; 3 Facultad de Ciencias, Escuela de Química y Farmacia, Universidad San Sebastián, Puerto Montt, Chile; Montana State University, Bozeman, Montana, USA

**Keywords:** wastewater irrigation, agricultural soils, soil microbiome, pollution, pathogens, Mezquital Valley, biogeochemical cycling, long-term irrigation, microbial diversity

## Abstract

The Mezquital Valley is a unique site for studying the gradual effects of wastewater irrigation on agricultural soils. We report metagenomes from soils irrigated for 50 years and their corresponding irrigation water. Potentially pathogenic bacteria dominated the wastewater, while the soil harbored a diverse community mainly involved in biogeochemical cycling.

## ANNOUNCEMENT

The Mezquital Valley is an ideal site for studying wastewater irrigation, showing gradual effects on agricultural soils, as the region contains plots that have been irrigated for periods ranging from months to more than 100 years (1). Wastewater irrigation represents a major selective pressure on soil microbiomes (2, 3), due to the continuous input of contaminants into the soils. Currently, little is known about the long-term genetic influence of wastewater irrigation on soil microbiomes.

Soils irrigated with wastewater for 50 years (irrigated soil, IS) were sampled in March 2020 from three agricultural plots. In each plot, four longitudinal transects were established, and superficial soil cores (0–10 cm; 2.6 cm diameter) were collected every 10 m, resulting in 120 cores per plot. Cores from each plot were homogenized to obtain one composite sample per plot (three composite samples in total). Wastewater samples (wastewater, WW) were collected from the Alto Ajacuba distribution canal using a glass sampler in November 2024. Three independent samples were collected in sterile bottles. Sampling-site coordinates are listed in Table 1. Wastewater and soil samples were transported to the laboratory at 4°C. Soil samples were stored at −20°C until DNA extraction, whereas wastewater samples were individually filtered through 0.22 µm nitrocellulose membranes.

**TABLE 1:** Summary of sequencing statistics and assembly quality metrics

Metric	Wastewater (WW)	Soil irrigated (IS) for 50 years
Coordinates of sampling sites	20°02′21.0″ N, 99°11′51.4″ W	Plot 1: 20°02′21.49″ N, 99°11′56.38″ W Plot 2: 20°02′18.10″ N, 99°11′59.29″ W Plot 3: 20°02′23.14″ N, 99°11′54.40″ W
Total reads	77,744,638	83,011,088
Total bases (Gbp)	5.8	6.2
Total sequences	38,872,319	41,505,544
Number of contigs	463,615	383,706
Total length (bp)	538,924,338	351,698,946
*N*_50_	1,262	898
*L*_50_	99,375	115,147
GC (%)	45.4	64.95
Bacteria	93.292	91.236
Archaea	0.975	0.746
Eukarya	5.546	7.961
Viruses	0.187	0.057
BioProject	PRJNA847174	PRJNA847174
BioSample	SAMN53072435	SAMN53072436
Raw sequencing data (SRA)	SRR35939818	SRR35939817
Assembled metagenomes (GenBank assembly accession)	JBXTSL000000000	JBXTSM000000000

**Fig 1:**
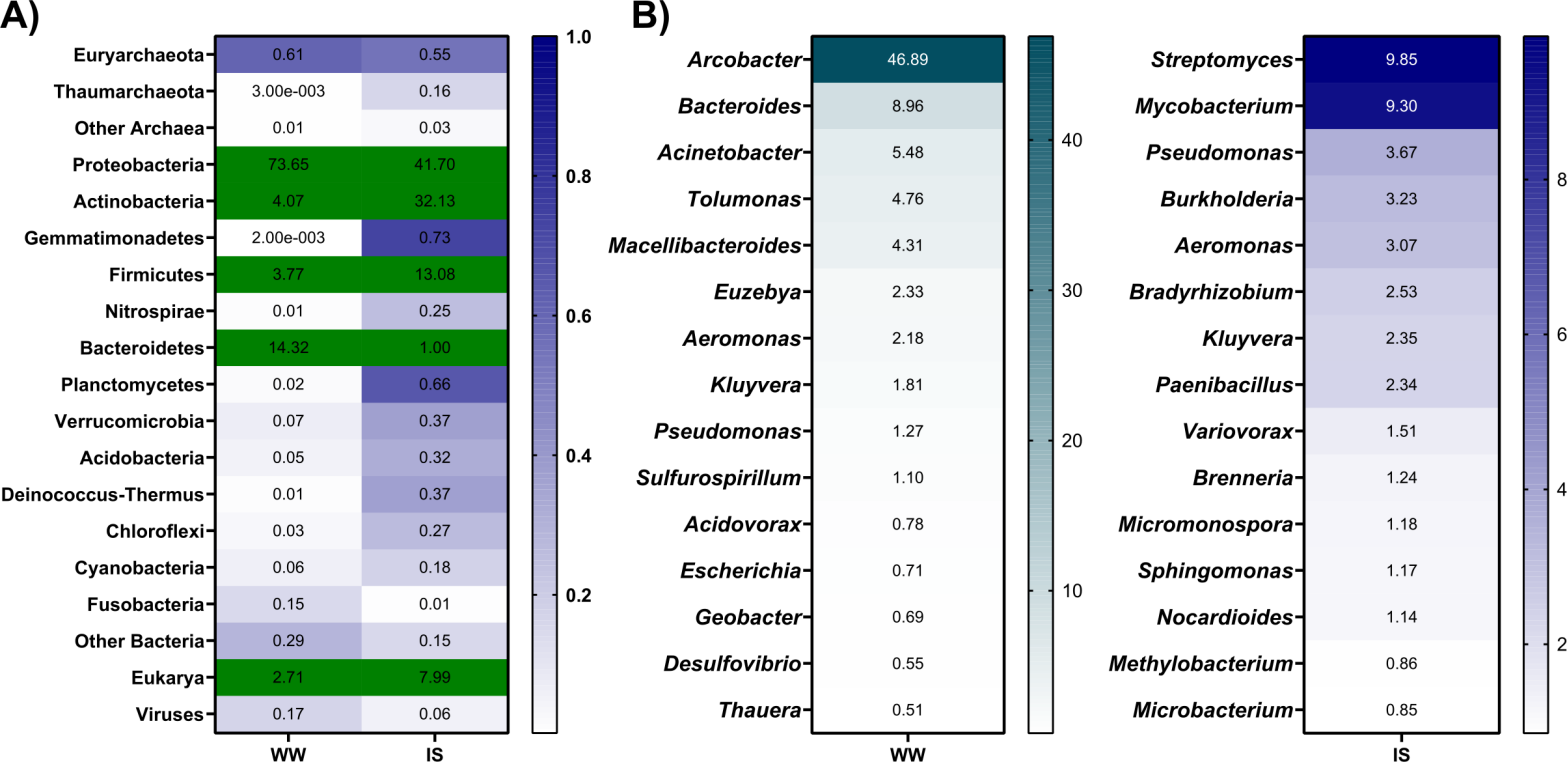
Heatmap of the relative abundances of the most abundant taxonomic groups identified in the analyzed metagenomes of wastewater (WW) and irrigated soil (IS) based on Kraken2 taxonomic assignment. (**A**) Phylum-level abundances. (**B**) Genus-level abundances.

DNA was extracted independently from the three composite soil samples (one per plot) and the three wastewater samples using the DNeasy PowerSoil Pro Kit and DNeasy PowerWater Kit (Qiagen), following the manufacturer’s instructions. DNA was quantified and pooled in equimolar proportions to generate one composite DNA sample for IS and one for WW. These two composite DNA samples were sequenced. Libraries were prepared using the TruSeq Nano DNA Kit (Illumina, USA). Paired-end sequencing (2 × 150 bp) was performed on an Illumina NovaSeq platform (Macrogen, South Korea). Quality control of the metagenomic reads was assessed using FastQC v0.12.1 (4), and the reads were filtered for quality (scores of ≥Q20) and adaptor sequences using Cutadapt v5.2 (5) and Trimmomatic v0.39 (6). Filtered reads were assembled *de novo* using MEGAHIT v1.2.9 using default k-mer sizes (7). Assembly quality was assessed with QUAST v5.3.0 (8), and assembly statistics are summarized in Table 1. Taxonomic classification of contigs was performed using Kraken2 v2.17.1 (9) with the core_nt database (v2024-09-04; downloaded 2025-02-05T204609Z) (Fig. 1A and B). The metagenomes were annotated with Prokka v1.14.6 using default parameters (10).

The WW metagenome was dominated by bacterial genera associated with clinical concern, including *Arcobacter*, *Campylobacter*, *Helicobacter*, and *Acinetobacter* (11–14). In contrast, the IS metagenome was enriched in soil-associated bacteria involved in nutrient cycling, such as *Mycobacterium*, *Streptomyces*, and *Pseudomonas* (15, 16) (Fig. 1B). Euryarchaeota represented the most abundant archaeal phylum in both metagenomes (17, 18), whereas eukaryotic and viral sequences were detected at lower relative abundances (Fig. 1A).

This metagenomic approach offers insights into the influence of wastewater on the genetic composition of irrigated soils in the Mezquital Valley. A chronosequence of soils irrigated in different time periods will help to understand the long-term effects of wastewater irrigation.

## Data Availability

Reads are available under SRA accession numbers SRR35939818 (wastewater) and SRR35939817 (soil). Assemblies are available under GenBank accession numbers JBXTSL000000000 (wastewater) and JBXTSM000000000 (soil); annotations are available at https://doi.org/10.6084/m9.figshare.31043680.
